# Addiction Therapeutic Communities in Saudi Arabia: An Evaluation of the Resident Retention Rates and Their Correlation With the Type of Drug Used

**DOI:** 10.7759/cureus.69045

**Published:** 2024-09-09

**Authors:** Abdulaziz Alshomrani

**Affiliations:** 1 Department of Internal Medicine, College of Medicine, University of Bisha, Bisha, SAU

**Keywords:** addiction treatment, deaddiction dropout, recovery, rehabilitation, retention, saudi, therapeutic community

## Abstract

Background: Long-term retention is a reliable, well-studied factor associated with enhanced outcomes in addiction therapeutic communities (ATCs). The aim of this study was to estimate resident retention rates of ATCs in Saudi Arabia at three and six months, completion of therapy, and early dropout, and investigate their correlation with the type of drug used and other social variables.

Material and methods: This was a cohort retrospective study and data of all residents admitted to all Saudi ATCs since their establishment in 2000 through September 2014 were collected from their files. There were five Saudi ATCs during the study period. The date of admission, discharge date, socio-demographics, and drug use were reported. Retention rates at three and six months and dropouts in the first week were calculated, and the correlation with the type of drug used was studied using multinomial binary logistic regression analysis.

Results: Out of a total of 2050 files, 2003 were suitable for analysis. All residents were male adults. The retention rate for three and six months was 45% and 28%, respectively, and 8.3% dropped out in the first week. The median duration of stay was 77 days. Unemployment and being a student were associated with the completion of treatment. The type of drug used showed no significant correlation with retention rates or dropouts.

Conclusion: Three-month retention, treatment completion, and dropout within the first week were reasonable, comparable, and consistent with reported rates worldwide. These rates can be considered an indicator of successful Saudi ATC programs. The type of drug used does not make a difference in retention and dropout rates in the present study, which is in line with the ATC management system that does not view the type of drug as a main treatment outcome modifier.

## Introduction

It is well known that addiction is a complex chronic disorder with frequent relapses affecting physical, psychological, and social health, which requires long continuous management to address the various biological, psychological, social, and spiritual needs [1]. One of the main intervention models that fulfills these needs is the therapeutic community model. An addiction therapeutic community (ATC) is a long-term residential treatment model that addresses these different aspects of patients' needs [2]. It is a drug-free residential treatment facility where 'community as method' is the cornerstone approach [2]. In this treatment model, addiction is a sign of disorganized personal life management, and stopping drug use is a manifestation of personal recovery that involves other physical, psychosocial, and spiritual aspects [2].

ATCs are effective in improving sobriety and reducing drug misuse, unemployment, criminalities, recidivism, relationship conflicts, homelessness, psychiatric comorbidities, drug injections, and drug overdoses [3-13]. Furthermore, it has been proven superior to several other types of addiction treatment modalities [1-13]. Retention for longer duration is a consistent well-studied factor that is associated with better ATC outcomes [1,4,5,10,11,14-16]. It has been noted that residents who have stayed for three months or more in ATC services have better outcomes than those who drop out earlier, which allowed some studies to consider a stay of 90 days as crucial [3,16]. The National Institute on Drug Abuse (NIDA) admits that treatment programs that last less than three months may not be successful in achieving intended outcomes [16].

A variety of factors linked to the retention of residents have been studied [7,8,15,17]. These factors can be divided into factors related to patient characteristics and factors related to the treatment program and its process [15]. Young age, unemployment, low education, severe drug use, and previous treatment history are well-known factors associated with early dropout [18]. In contrast, factors like high education, employment, legal pressure, enhancing residents' motivation, and involvement of the family and social network in treatment are related to resident's retention improvement [5,19-24]. Furthermore, Meier and Best noted that a not-too-large, well-scheduled treatment program with healthy and not-so-stressful organized activities with appropriate individual counseling correlates with a high completion rate of the treatment program [19]. However, the relationship between treatment retention and drug type, presence of comorbidities, history of incarceration, race, and gender have often shown conflicting and contradictory results [14]. This study aimed to estimate retention rates in ATCs in Saudi Arabia, study the drug use patterns and characteristic factors associated with early dropout and long-term retention, and investigate their correlation with the type of drug used.

This article was previously posted on the Research Squre Preprint server in December 2020.

## Materials and methods

Population and data source

The study included data from records of all discharged residents of ATCs in Saudi Arabia from the time ATCs were established (2000) up to September 2014. The reason for discharge could be related to completing the treatment program, discharge against treatment advice, dismissal from the program due to breaking the roles, or any other cause. Discharged residents with missing data were excluded due to incomplete records. The study followed the Good Clinical Care guidelines and guidelines of the Declaration of Helsinki. The study was approved by the Institutional Review Board of the College of Medicine at Al-imam Mohammad lbn Saud Islamic University (approval number: HAPO-01-R-010).

During the study period, there were only five addiction ATCs in the Kingdom of Saudi Arabia, and all of them were included. Two of the ATCs were in Dammam City and one each was located in Riyadh, Jeddah, and Taif. The first ATC in Saudi Arabia was started in 2000 and was part of the Alamal Complex for Mental Health in Dammam. However, it took almost a decade to establish the next ATC. The other four ATCs were established between 2009 and 2013. Three of the five ATCs studied are governmental, while the other two were operated by non-governmental societies [24]. All of them were traditional ATCs with almost similar treatment and rehabilitation programs [24]. The usual recommended length of stay was six to nine months. Therefore, those who stayed six months or more were defined as treatment program completers. All these ATCs were for males and did not accept individuals aged less than 20 years or involuntary admissions.

Two paramedical staff collected data under the author's supervision from the patient charts and records using comprehensive forms that included demographic information, number of admissions, diagnoses, admissions dates, lengths of stays, and addicted substances. The data was collected between July and September, 2015. The author visited each ATC, assigned two paramedical staff to each ATC, and trained them on collecting data from resident files and records and filling in previously structured forms. All files were included except those with missing admission and discharge dates.

Statistical analysis

Data were recorded into Microsoft Excel sheets (Microsoft Corporation, Redmond, Washington, United States) and exported into IBM SPSS Statistics for Windows, Version 21.0 (Released 2012; IBM Corp., Armonk, New York, United States) for further analysis. Descriptive statistics were used to represent the data. Quantitative variables were expressed as mean, standard deviation (SD), median, and range, while categorical data were expressed as frequency and percentage and analyzed using the Chi-square test. P-value <0.05 was considered significant, and variables with p-value <0.05 were entered into multinomial binary logistic regression analysis. Therapeutic communities were labeled by numbers rather than their names for convenience.

## Results

Out of a total of 2050 files, data was collected from 2003 patients who were discharged from any of the five Saudi ATCs before the end of September 2014. General characteristics of the subjects are shown in Table 1. All residents were adult males since there were no ATCs for females or adolescents in Saudi Arabia at the time of the study. More than two-thirds of patients were younger than 40 years of age. Forty-three percent belonged to ATC 3. The majority were single (75.9%, n=1494), unemployed (68%. n=1259), and had intermediate or secondary school education (73%, n=1421 ).

**Table 1:** Demographic characteristics of the residents (N=2003) ATC: addiction therapeutic community

Variable	Frequency	Percentage
Age (years)		
20-29	721	36
30-39	723	36.1
40-49	437	21.8
>50	103	5.1
Residency		
ATC 1	511	25.5
ATC 2	147	7.3
ATC 3	859	42.9
ATC 4	26	1.3
ATC 5	459	22.9
Occupation		
Unemployed	1259	68.4
Student	48	2.6
Employed	517	28.1
Retired	16	0.9
Marital status		
Single	1494	75.9
Married	475	24.1
Education		
Illiterate	20	1
Primary	417	21.5
Intermediate	689	35.4
Secondary	732	37.7
University	86	4.4

Of the patients, 46% patients reported opioid use, 36% (n=721) hash, 34% (n=681) amphetamine, and 19% (n=381) reported alcohol misuse (Table 2). Most of the patients were admitted once (92%, n=1843), 6.7% (n=143) were admitted twice, and only 1.4% (n=28) were admitted more than two times.

**Table 2:** Distribution of patients (N=2003) according to drug use, number of admissions, and duration of stay

Variable	Frequency	Percentage
Type of drugs used		
Alcohol	135	7
Amphetamine	223	11.5
Hash	260	13.4
Opioid	723	37.2
Amphetamine and Alcohol	56	2.9
Hash and Amphetamine	237	12.2
Hash and Alcohol	72	3.7
Hash and Opioid	19	1
Multiple (>3 drugs)	207	10.7
Number of admissions		
One	1839	91.9
Two	126	6.7
Three	14	0.8
Four	9	0.4
Five	3	0.1
Duration of stay		
1-7 days	166	8.3
8-90 days	933	46.6
91-180 days	345	17.2
More than 180 days	558	27.9
Median	77	

Since the program's minimum duration was six months, 28% (561) of the patients were considered treatment completers. Dropouts in the first week were 8.3% (166) of all admissions, and almost 45% (901) stayed for over three months. The median duration of stay was 77 days. The first-week dropout was associated only with staying at ATC 2 and ATC 3. As demonstrated in Table 3, univariate analysis shows age, place of residency, and type of drug used significantly associated with retention for three months. However, these associations were insignificant after multiple regression analyses except residency in ATC 1, ATC 2, and ATC 4, which were less likely to stay for more than three months as shown in Table 4. After adjusting for other confounders, multiple regression analysis excluded age, and type of drug used, and confirmed being students to be associated with treatment completion. On the other hand, admission to ATC 1 and ATC 2 proved to be significantly associated with treatment program incompletion as demonstrated in Table 5. 

**Table 3:** Distribution of factors associated with first-week dropout, three-month retention, and six-month retention ATC: addiction therapeutic community

Characteristics	First-week Dropouts	P-value	Three-month Retention	P-value	Six-month Retention	P-value
	Frequency (Percentage)		Frequency (Percentage)		Frequency (Percentage)	
Age (years)		0.279		0.001		0.000
20-29	61 (8)		287 (40)		177 (25)	
30-39	71 (10)		327 (45)		184 (25)	
40-49	25 (6)		225 (51)		151 (35)	
>50	8 (8)		57 (55)		43 (42)	
Residency		0.000		0.001		0.000
ATC 1	53 (10)		268 (52)		184 (36)	
ATC 2	7 (5)		94 (64)		62 (42)	
ATC 3	35 (4)		340 (40)		214 (25)	
ATC 4	51 (9)		17 (65)		3 (12)	
ATC 5	66 (14)		184 (40)		95 (21)	
Occupation		0.258		0.61		0.009
Unemployed	89 (7)		557 (44)		184 (36)	
Student	3 (6)		17 (35)		62 (42)	
employed	48 (9)		263 (51)		214 (25)	
Retired	0 (0)		8 (50)		3 (12)	
Marital status		0.053		0.067		0.175
Single	111(7)		659 (44)		405 (27)	
Married	50 (11)		227 (48)		144 (30)	
Education		0.881		0.481		0.455
Illiterate	1 (5)		5 (25)		3 (15)	
Primary	35 (8)		187 (45)		111 (27)	
Intermediate	54 (8)		317 (46)		206 (30)	
Secondary	56 (8)		346 (47)		213 (29)	
University	9 (10)		37 (43)		22 (26)	
Type of drug used		0.782		0.039		0.031
Alcohol	10 (7)		51 (38)		30 (22)	
Amphetamine	18 (8)		106 (4)		64 (29)	
Hash	27 (10)		109 (42)		58 (22)	
Opioid	48 (7)		359 (50)		230 (32)	
Amphetamine and Alcohol	5 (9)		25 (45)		17 (30)	
Amphetamine and Hash	18 (8)		88 (37)		54 (23)	
Hash and Alcohol	6 (8)		29 (40)		18 (25)	
Opioid and Hash	2 (11)		8 (42)		4 (21)	
Multiple (>3drugs)	21 (10)		105 (51)		68 (33)	
Number of admissions		0.741		0.177		0.206
One	155 (8)		839 (46)		524 (46)	
Two	9 (7)		52 (39)		27 (47)	
Three	2 (13)		8 (50)		5 (48)	
Four	0 (0)		4 (44)		2 (49)	
Five	0 (0)		0 (0)		0 (50)	

**Table 4:** Multinomial binary logistic regression analysis of all predictors of first-week dropout and three-month retention with P < 0.05 on univariate analyses ATC: addiction therapeutic community; df: degree of freedom

		Std. Error	Wald	df	OR (95%CI)	P-value
First-week dropout						
Residency						
ATC 1		0.203	2.674	1	1.393 (.936-2.073)	0.102
ATC 2		0.414	7.552	1	3.122 (1.386-7.032)	0.006
ATC 3		0.225	33.874	1	3.703 (2.383-5.755)	0.000
ATC 4		0.523	0.705	1	0.645 (.232-1.796)	0.401
ATC 5		.	.	0	.	.
Three-month retention						
Age category		0.577	0.565	1	1.543 (0.498-4.780)	0.452
Residency						
ATC 1		0.168	13.698	1	0.538 (0.387-0.747)	0.000
ATC 2		0.219	19.185	1	0.383 (0.249-0.589)	0.000
ATC 3		0.144	0.047	1	1.032 (0.779-1.367)	0.829
ATC 4		0.439	5.638	1	.353 (0.149-0.834)	0.018
ATC 5		.	.	0	.	.
Type of drug used						
Alcohol		0.246	2.816	1	1.512 (0.933-2.450)	0.093
Amphetamine		0.218	0.315	1	0.885 (0.577-1.357)	0.575
Hash		0.206	0.284	1	1.116 (0.745-1.673)	0.594
Opioid		0.184	0.289	1	0.906 (0.632-1.299)	0.591
Amphetamine and Alcohol		0.330	0.047	1	0.931 (0.488-1.776)	0.828
Amphetamine and Hash		0.214	1.423	1	1.292 (0.848-1.966)	0.233
Hash and Alcohol		0.294	0.336	1	1.186 (0.666-2.109)	0.562
Opioid and Hash		0.549	0.146	1	1.233 (0.420-3.618	0.703
Multiple (>3 drugs)		.	.	0	.	.

**Table 5:** Multinomial binary logistic regression analysis of all predictors of six-month retention with P < 0.05 on univariate analysis ATC: addiction therapeutic community; df: degree of freedom

Six-months Retention	Std. Error	Wald	df	Odds ratio (95%CI)	P-value
Age category	0.695	2.625	1	3.083 (0.790-12.040)	0.105
Residency					
ATC 1	0.184	14.929	1	0.492 (0.343-0.705)	0.000
ATC 2	0.227	12.607	1	0.447 (0.286-0.697)	0.000
ATC 3	0.162	0.007	1	0.987 (0.718-1.357)	0.934
ATC 4	0.64	1.854	1	2.391 (0.682-8.388)	0.173
ATC 5	.	.	0	.	.
Occupation					
Unemployed	0.634	0.604	1	1.636 (0.473-5.665)	0.437
Student	0.795	4.44	1	5.338 (1.124-0.352)	0.035
Employed	0.638	0.085	1	1.205 (0.345-4.210)	0.77
Retired	.	.	0	.	.
Type of drug used					
Alcohol	0.246	2.816	1	1.441 (0.845-2.459)	0.18
Amphetamine	0.218	0.315	1	0.796 (0.502-1.263)	0.333
Hash	0.206	0.284	1	1.105 (0.707-1.727)	0.661
Opioid	0.184	0.289	1	0.881 (0.598-1.298)	0.522
Amphetamine and Alcohol	0.33	0.047	1	0.682 (0.344-1.353)	0.274
Amphetamine and Hash	0.214	1.423	1	1.093 (0.688-1.738)	0.706
Hash and Alcohol	0.294	0.336	1	1.017 (0.540-1.918)	0.957
Opioid and Hash	0.549	0.146	1	1.028 (0.309-3.422)	0.964

## Discussion

Three-month retention rates of 45% in Saudi ATCs, as found in this study, are acceptable and match the documented rates worldwide, and are consistent with previous studies. Most studies of long-term ATCs reported a three-month retention rate between 30% and 50% [3,9,17]. Gossop and his colleagues studied retention rates in 11 long-term residential programs in England and reported a 40% retention rate for the same period [10]. In other studies, the retention rate for three months was 25%, 31%, and 32% among intravenous drug injectors [25], amphetamine users, and all other drug users [26], respectively. On the contrary, Mulder and his colleagues reported a rate of 57% [20]; however, the study population was small, and 71% came to treatment due to court orders and not of their free will as in our study population. Furthermore, the program completion rate was 28%, which lay in the middle of the ranges of other studies, 9-56% [8].

Our study population was similar in age, employment, gender, and marital status to the studies mentioned earlier. Most of the drugs used are opioids, as in some other studies [10,17]. The median stay was 77 days compared to 70 days reported in English long-term residential treatment facilities [10] and 39 days in a prospective Australian study [14]. Although Saudi ATCs followed the same program structure and showed no differences in essential ATC treatment elements [24], the significant differences in retention rates need to be explored.

Being a student was associated with treatment completion and residents of some specific ATCs were found to be more prone to drop out before completing the program. Interestingly, the type of drug used was not associated with dropout and retention at one week, three months, or six months, matching the findings of other studies [20,27]. ATC sees the addiction as one of the manifestations of personal disorganization. So the type of drug is not a main treatment outcome modifier [2]. The finding of our study thus agrees with the ATC view. The first-week dropout in the current study was 8.3%, which was better than most similar studies where dropout was 17-20%; however, the high dropout makes these few days critical, requiring more targetted and specially tailored approaches. As in other periods, retention was significantly different between the Saudi ATCs. This means mean therapeutic community paradigm implementation may not be on the same level of authenticity.

Limitations

This is a retrospective study that depends on the documentation authenticity of the file's data. All the ATCs were limited to adult male residents which interfered with the result generalization to adolescents, females, and modified ATCs. Drug use patterns and trends keep changing every few years and sometimes within a year; thus the restricted timeframe of the study may have an impact on the applicability of the results.

## Conclusions

The retention and dropout rates of Saudi ATCs were more or less similar to those registered worldwide reflecting a good indicator of their effectiveness. This may support a recommendation to establish further ATCs in other cities and regions since they were limited mainly to four cities. However, the significant retention rate differences among different ATCs may need to be explored further to determine the factors that may play roles in these differences. The type of drug used does not make a difference in retention and dropout rates, which may support the ATC view that does not see the type of drug as a main treatment outcome modifier. A prospective cohort study is needed to affirm the outcomes of this study.
